# Association of maternal serum concentrations of 2,2', 4,4'5,5'-hexachlorobiphenyl (CB-153) and 1,1-dichloro-2,2-bis (p-chlorophenyl)-ethylene (p,p'-DDE) levels with birth weight, gestational age and preterm births in Inuit and European populations

**DOI:** 10.1186/1476-069X-9-56

**Published:** 2010-09-06

**Authors:** Bogdan J Wojtyniak, Daniel Rabczenko, Bo AG Jönsson, Valentyna Zvezday, Henning S Pedersen, Lars Rylander, Gunnar Toft, Jan K Ludwicki, Katarzyna Góralczyk, Anna Lesovaya, Lars Hagmar, Jens Peter Bonde

**Affiliations:** 1 Department-Centre of Monitoring and Analyses of Population Health, National Institute of Public Health - National Institute of Hygiene, Warsaw, Poland; 2 Division of Occupational and Environmental Medicine, Lund University, Lund, Sweden; 3 Laboratory of Human Reproduction, Kharkiv State Medical University, Kharkiv, Ukraine; 4 Centre for Arctic Environmental Medicine, Nuuk, Greenland; 5 Department of Occupational Medicine, Aarhus University Hospital, Aarhus, Denmark; 6 Department of Environmental Toxicology, National Institute of Public Health - National Institute of Hygiene, Warsaw, Poland; 7 www.inuendo.dk

## Abstract

**Background:**

Epidemiological studies on the association between maternal exposure to persistent organic pollutants (POPs) and fetal growth alteration report inconsistent findings which weights in favor of additional studies.

**Methods:**

Blood samples were collected from interviewed pregnant women in Greenland (572), Kharkiv (611) and Warsaw (258) and were analyzed for CB-153 and p,p'-DDE by gas chromatography-mass spectrometry. Data on birth weight, gestational age and preterm birth were obtained for 1322 singleton live births. We examined the association between natural log-transformed serum POPs concentration and birth weight and gestational age using multiple linear regression and the association with prematurity using logistic regression controlling for potential confounding factors.

**Results:**

The median serum concentrations of CB-153 and p,p'-DDE were for Inuit mothers 105.6 and 298.9, for Kharkiv mothers 27.0 and 645.4 and for Warsaw mothers 10.7 and 365.2 ng/g lipids, respectively. Increase in CB-153 concentration by one unit on the log scale in Inuit mothers serum was associated with significant decrease in infant birth weight of -59 g and gestational age by -0.2 week. Decreases observed in the cohorts in Kharkiv (-10 g and -0.1 week) and in Warsaw (-49 g and -0.2 week) were not statistically significant. Increase in p,p'-DDE concentration by one unit on the log scale was associated with a statistically significant decrease in infant birth weight of -39.4 g and -104.3 g and shortening of gestational age of -0.2 week and -0.6 week in the Inuit and Warsaw cohorts, respectively. In the Kharkiv cohort decrease in birth weight (-30.5 g) was not significant, however a shortening of gestational age of -0.2 week per increase in p,p'-DDE concentration by one unit on the log scale was of the borderline significance. There was no significant association between CB-153 and p,p'-DDE concentrations and risk of preterm birth however, in all cohorts the odds ratio was above 1.

**Conclusions:**

*In utero *exposure to POPs may reduce birth weight and gestational age of newborns however, new insights as to why results vary across studies were not apparent.

## Background

Persistent Organic Pollutants (POPs) are chemicals that persist in the environment, accumulate in high concentrations in fatty tissues and are bio-magnified through the food-chain. They constitute a potential environmental hazard causing possible long-term risks to human health. They are border-crossing substances transported long distance, predominately by air and critical concentrations have been reached in some regions, even in places where they have never been produced or used. POPs have been detected in human blood, adipose tissue and breast milk all over the world. Chlorinated hydrocarbon pesticides are not presently recommended for the use in agriculture, and in public health programs only a few persistent chlorinated hydrocarbon pesticides are still being used in a few countries, especially DDT, in vector disease control. Similarly, PCBs widely used by the industry in the past and now ultimately banned for at least two decades are still transported through different environmental media.

Although there has been a growing concern about the effect of POPs on human fetal development and birth outcomes, variation in results across studies remains unexplained. Epidemiological findings regarding association of POPs such as polychlorinated biphenyls (PCBs) and pesticides such as DDT with human fetal growth alteration are inconsistent [1-4]. There are several studies demonstrating that exposure to PCBs at ordinary environmental levels was related to a reduction in infant birth weight [5-8] however, there are also studies showing little evidence to support such an association [9-12]. In all those studies PCB exposure was assessed from maternal or cord blood. Similar inconsistency is present in the studies with respect to gestational age or premature births [3,11-14]. Not less inconsistent are the results concerning an association between exposure to the major, persistent, and antiandrogenic DDT metabolite 1,1-dichloro-2,2-bis (*p-*chlorophenyl)-ethylene (p,p'-DDE) and birth weight and length of gestation [11,15,16].

The present study stems from a collaborative research project funded by EU (Inuendo; http://www.inuendo.dk) aiming to enlighten the impact of dietary POPs on human reproductive function in an epidemiological setting of varying POPs exposure. The Inuendo project study population has been established in three European countries - Sweden, Poland (Warsaw) and Ukraine (Kharkiv) together with a population of Inuits from Greenland. Since we were able to follow-up most of the participating pregnant women from Greenland, Warsaw and Kharkiv till pregnancy termination it gave us an opportunity to analyze some characteristics of the newborns as an additional measurements of POPs impact on human reproduction. We have chosen to use the PCB congener, 2,2', 4,4'5,5'-hexachlorobiphenyl (CB-153) in serum as a biomarker for POPs exposure because of its very high correlations with the total PCB concentration [17-19]. Another relevant biomarker is p,p'-DDE. Detailed analysis of the inter-population variation in CB-153 and p,p'-DDE serum concentrations and of the determinants of this variation in the study populations has been given elsewhere [20]. The aim of this study was to investigate the association between maternal serum level of CB-153 and p,p'-DDE and birth weight, length of gestation and risk of preterm birth. The number of low birth weight (below 2500 g) infants was too small for a separate analysis of the risk of this pregnancy outcome.

## Methods

**Figure 1:**
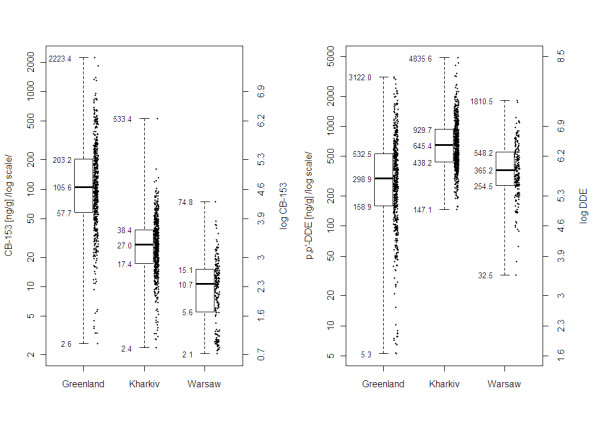
**Distribution of maternal serum concentration of CB-153 and p,p'-DDE in the study populations (left-hand side axis has a logarithmic scale for untransformed concentration values, right-hand side scale is linear for log-transformed concentration values)**.

Between June 2002 and May 2004 we recruited pregnant women and their male spouses in Greenland, Kharkiv (Ukraine) and Warsaw (Poland) for interviews and blood sampling. A general criterion for eligibility was that the participants were born in the country of study and were at least 18 years of age. A detailed description of the recruitment process, population characteristics and data collection has been given elsewhere [20,21]. In brief, the target population consisted of pregnant women who visited antenatal health care units. In Greenland Inuit women lived in 19 municipalities and settlements throughout the country, out of 665 eligible approached 35 refused and 32 were inaccessible. Thus 598 (89.9%) were interviewed and 572 (86.0%) also donated a blood sample. In Ukraine the women lived in Kharkiv and surrounding villages and visited one of three maternity hospitals or eight antenatal clinics in the city. Altogether 2478 pregnant women were informed about the project and asked to participate of whom 632 (25.8%) were interviewed and 611 (24.7%) donated a blood sample. One of the reasons that a large number of pregnant women refused to participate was concern that the collection of blood sample would imply a risk for the pregnancy and the baby - in particular when anemia had been detected during pregnancy. Demographic and reproductive information obtained from 605 of those women who declined participation in the study show that the average age in the group (22.8 years) was slightly lower than among those who participated (24.9 years), while the average number of children in both the groups was similar (1.1 versus 1.2 among those with at least one child). In Poland the women lived mostly in Warsaw and its suburbs and visited the obstetric out-patient clinic of a large Gynecological and Obstetric Hospital or physicians at a collaborating hospital also in Warsaw. Altogether 690 women were informed about the project and invited to participate of whom 472 (68.4%) were interviewed and 258 (37.4%) donated a blood sample. In total 1441 women were interviewed and donated blood and 1322 of them delivered a singleton infant for whom all required information (gestational age, sex and birth weight) were available. Analyzed sample size, pregnancy outcomes and levels of exposure chemicals are presented in Table 1 and Figure 1.

**Table 1:** Analyzed sample size, levels of pregnancy outcomes and of exposure chemicals

	Greenland	Kharkiv	Warsaw
Mothers approached	665 (100.0%)	2478 (100.0%)	690 (100.0%)
Mothers interviewed	598 (89.9%)	640 (25.8%)	472 (68.4%)
Blood samples	572 (95.7%)	611 (95.5%)	258 (54.7%)
CB-153 [ng/g lipid] ^1^	105.4 (± 2.8)	25.7 (± 1.9)	9.0 (± 2.1)
p,p'-DDE [ng/g lipid] ^1^	273.8 (± 2.9)	653.3 (± 1.8)	356.8 (± 1.9)

Live births analyzed	547 (82.3%)	577 (23.3%)	198 (28.7%)
Preterm births	28 (5.1%)	12 (2.1%)	12 (6.1%)
Gestational age [weeks]	39.6 (± 1.9)	39.1 (± 1.2)	39.2 (± 1.8)
Birth weight [grams] ^2^	3582 (± 611)	3273 (± 438)	3453 (± 506)

^1^geometric mean

^2^full term births

The Inuit and Ukrainian women were earlier in their pregnancies at the time of interview and blood sample collection (on average 24 weeks pregnant) than the Polish women (33 weeks pregnant). Through the interview we obtained information on the maternal demographic and social factors and time varying characteristics such as weight, smoking, alcohol drinking and occupational exposures were obtained for the period of attempt to conceive (Table 2).

**Table 2:** Characteristics of the study cohorts - potential confounding factors

		Greenland	Kharkiv	Warsaw
Age [years]		26.8 (± 6.1)	6.1 (± 25)	25 (± 4.8)

Pre-pregnancy BMI [kg/m^2^]		24.5 (± 4.3)	4.3 (± 21.6)	21.6 (± 3.2)

Education	left before 15 or 15-17	227 (41.5%)	156 (27%)	-
	left at age 18	46 (8.4%)	144 (25%)	44 (22.2%)
	continued above age 18	211 (38.6%)	207 (35.9%)	150 (75.8%)
	missing	63 (11.5%)	70 (12.1%)	4 (2.0%)

Marital status	married	114 (20.8%)	322 (55.8%)	160 (80.8%)
	living as married	323 (59.0%)	195 (33.8%)	32 (16.2%)
	living alone	55 (10.1%)	51 (8.8%)	6 (3.0%)
	missing	55 (10.1%)	9 (1.6%)	0 (0.0%)

Smoking^1^	never smoker	64 (11.7%)	368 (63.8%)	129 (65.2%)
	smoker	65 (11.9%)	78 (13.5%)	32 (16.2%)
	ex-smoker	418 (76.4%)	131 (22.7%)	37 (18.7%)

Exposure to other people's cigarette smoke^1^	no	146 (26.7%)	110 (19.1%)	71 (35.9%)
	yes	390 (71.3%)	455 (78.9%)	126 (63.6%)
	missing	11 (2.0%)	12 (2.1%)	1 (0.5%)

Alcohol drinks^1^	less than 14/week	519 (94.9%)	576 (99.8%)	197 (99.5%)
	14 and more/week	28 (5.1%)	1 (0.2%)	1 (0.5%)

Occupational exposure^1^	no	539 (98.5%)	497 (86.1%)	172 (86.9%)
	yes	8 (1.5%)	80 (13.9%)	26 (13.1%)

Previous pregnancies	0	63 (11.5%)	320 (55.5%)	148 (74.7%)
	1	83 (15.2%)	127 (22.0%)	34 (17.2%)
	2+	395 (72.2%)	107 (18.5%)	9 (4.5%)
	missing	6 (1.1%)	23 (4.0%)	7 (3.5%)

Newborn sex	Boy	295 (53.9%)	300 (52.0%)	96 (48.5%)
	Girl	292 (53.4%)	277 (48.0%)	102 (51.5%)

^1^during the period of attempt to conceive

The study was approved by local ethical committees and all subjects signed an informed consent.

### Determination of CB-153 and p,p'-DDE in serum

All analysis of CB-153 and p,p'-DDE in serum were performed in the Department of Occupational and Environmental Medicine in Lund, Sweden and the procedures were described in detail elsewhere [20,22,23]. Briefly, serum concentrations of CB-153 and p,p'-DDE were analyzed by gas chromatography mass spectrometry following solid phase extraction. The relative standard deviations, calculated from samples analyzed in duplicate at different days, was 18% at 0.1 ng/mL (n = 990), 10% at 0.5 ng/mL (n = 990) and 10% at 2 ng/mL (n = 990) for CB-153 and 11% at 1 ng/mL (n = 1058), 8% at 3 ng/mL (n = 1058) and 7% at 8 ng/mL (n = 1058) for p,p'-DDE.

The determination limits were 0.05 ng/mL for CB-153 and 0.1 ng/mL for p,p'-DDE. In the case of non-detectable concentrations, the results were set at half of the determination limit. There were 69 such values for CB-153 and 10 for p,p'-DDE. The results were expressed on a lipid weight basis determined by enzymatic methods [20].

### Outcome measures

At the end of the field study the pregnancy outcome short questionnaire was filled in by medical personnel at the health centers where delivery took place using data from personal forms of pregnant women and the history of their delivery. Three outcomes were assessed in the analysis: 1) birth weight (in grams) analyzed only for full-term birth - 37 and more weeks of completed gestation; 2) gestational age (in weeks) defined as the duration between a self-reported date of the last menstrual period and the date of birth; and 3) preterm birth, before 37 week of gestation. The information from singleton live births were taken into account only. Of 1322 births 52 (3.9%) were born preterm while only 19 full term newborns had low birth weight (less than 2500 g) therefore we did not analyze this outcome variable. The mothers who were interviewed but were not included in the birth outcome analysis (26 in Greenland, 29 in Kharkiv and 214 in Warsaw) did not differ significantly from the analyzed group taking into account the socio-demographic variables that were considered as potential confounding factors.

### Statistical analysis

We examined the association between CB-153 and p,p'-DDE serum concentrations and birth weight and gestational age, respectively, using linear regression models and relation to prematurity using logistic regression models built for each country independently.

We assessed the shape of the relationship between POPs concentrations and outcome variables using generalized additive models with integrated smoothness estimation and derived number of the effective degrees of freedom (EDF) [24]. If the number of EDF was above one (thus relationship could be viewed as non-linear) we fitted a parametric model with natural log-transformed POP variable. Nonlinear association was found in 10 of 18 analyzed relationships. Next, using analysis of deviance [25], we compared fit of non-parametric and logarithmic models. In all the cases there were no significant differences between the fit. If dependence could be regarded as linear (EDF = 1) we checked if a linear model could be replaced by a logarithmic one. The comparison was based on R^2 ^in the case of ordinary regression and in terms of a generalized R^2 ^(computed as a ratio of deviance of the model to null deviance) in the case of logistic regression. Since values of the compared statistics were similar, we report estimates from the logarithmic model for easier comparability of the results. The effect point estimates are presented together with a 95% confidence interval (95% C.I.). Because analyzed concentrations of CB-153 and p,p'-DDE were log-transformed linear regression coefficients presented in the tables represent changes in the outcome variables (birth weight, gestational age) associated with increase in chemical concentration by one unit on the log scale. In the logistic regression analysis that we applied to premature birth, the effects of concentrations are presented as odds ratios associated with their twofold increase. In the case of natural-log transformed independent variable "x" odds ratio associated with change of this dependent variable from its value x_1 _to x_2 _is given by formula $e^{b_{1}\ln(\frac{x_{1}}{x_{2}})}$, where b_1 _is a regression coefficient. To show results in a generalized way, we applied common approach in such a situation and calculated odds ratio for the case when x_1_/x_2 _= 2 which is denoted as odds ratio associated with twofold increase of untransformed concentration.

To control for the confounding of the exposure-outcome association by other factors we built multivariate models taking into account such covariates as maternal age, body mass index (BMI) before pregnancy, education, marital status, smoking status, alcohol drinking, occupational exposure (to paints, solvents, fumes, engine exhaust), parity and also the newborn's sex. Covariates were included in the models in a stepwise forward fashion if the addition of each variable to the model changed the regression coefficient of the chemical serum concentration by at least 10% [26]. In the final step of the birth weight model we added gestational age, known to influence birth weight, to check to what extent birth weight reduction due to the POP exposure may be related to shortened gestational age.

The analyses were done using the S-PLUS 2000 [27] and R 2.9.2 [28] statistical programs. The term statistically significant in all performed analyses indicates a p-value of less than 0.05.

## Results

There were differences in the mean birth weight of singleton, live, full term newborns among the study cohorts (Table 1). The heaviest were newborns in Greenland and the lightest in Kharkiv. On the other hand preterm births were less frequent in Kharkiv than in the other two study groups. The Inuit mothers had a distinctly higher serum concentration of CB-153 as compared with mothers from Warsaw and Kharkiv (age-adjusted geometric mean ratio 12.7 and 3.8, respectively). The highest serum concentrations of p'p-DDE was observed in Kharkiv mothers (age-adjusted geometric mean ratio 2.52 and 2.02 when compared to Inuit and Warsaw mothers, respectively).

Overall, we found statistically significant relations between maternal serum concentration of CB-153 and birth weight and length of gestation only in the Inuit population (Table 3).

**Table 3:** Regression coefficients^1 ^and odds ratios^2 ^(OR) from unadjusted and multivariate linear and logistic regression models of maternal serum log CB-153 and birth weight, gestational age and premature birth

	*Birth weight (grams)*			*Gestational age (weeks)*			*Prematurity*		
	**Regression**	**95% C.I**.	**p**	**Regression**	**95% C.I**.	**p**	**OR**	**95% C.I**.	**p**
	**coefficient**			**coefficient**					

*Unadjusted^3 ^model*									
Greenland	-87.6	(-132.9; -42.3)	< 0.01	-0.2	(-0.4; 0.0)	0.02	1.21	(0.93; 1.58)	0.16
Kharkiv	-24.6	(-75.2; 26.0)	0.34	-0.2	(-0.4; 0.0)	0.05	1.43	(0.78; 2.63)	0.26
Warsaw	-45.6	(-129.1; 37.9)	0.29	-0.1	(-0.5; 0.3)	0.62	0.93	(0.55; 1.58)	0.79

*Multivariate model^3,4^*									
Greenland	-72.7	(-118.0; -27.4)	< 0.01	-0.2	(-0.4; 0.0)	0.02	1.14	(0.86; 1.52)	0.36
Kharkiv	-17.9	(-71.2; 35.4)	0.51	-0.1	(-0.3; 0.1)	0.12	1.28	(0.62; 2.65)	0.51
Warsaw	-61.6	(-152.3; 29.1)	0.19	-0.2	(-0.6; 0.2)	0.40	1.29	(0.65; 2.55)	0.47

*Multivariate model, with gestational age*									
Greenland	-59.2	(-100.6; -17.8)	0.01						
Kharkiv	-10.2	(-61.4; 41.0)	0.7						
Warsaw	-49.4	(-134.5; 35.7)	0.26						

^1^Per one unit increase of natural log CB-153

^2^Per twofold increase of untransformed CB-153

^3^Number of subjects in unadjusted/multivariate models: Greenland birth weight 519/519, gestational age 547/547, prematurity 547/547; Kharkiv birth weight 565/554, gestational age 577/577, prematurity 577/577; Warsaw birth weight 186/186, gestational age 198/198, prematurity 198/198

^4^Models for birth weight controlled for: smoking status (Greenland); age of mother, mother's BMI, gender of child, number previous pregnancies, mother's martial status, passive smoking (Kharkiv); age of mother, gender of child, mother's education status, mother's BMI (Warsaw);

Models for gestational age controlled for: none (Greenland); mother's age (Kharkiv); mother's education level, mother's age, previous pregnancies, exposition, alcohol drinking (Warsaw);

Models for prematurity controlled for: mother's education level, mother's age, martial status (Greenland); mother's education level, previous pregnancies, smoking status, marital status, mother's age (Kharkiv); mother's BMI, mother's age (Warsaw);

Increase in log CB-153 concentration was associated with statistically significant decrease in infants birth weight, however adjustment for confounding variables in a multivariate models reduced the effect preserving its significance.

It indicates that the possible influence of CB-153 exposure on birth weight of Inuit infants is in part mediated through a change in gestational age. This sequence is further corroborated by a significant albeit small reduction in gestational age in Inuit infants associated with increased CB-153 concentration in maternal blood. However, even in the Inuit population the risk of premature birth did not grow significantly with the increase in the serum concentration of CB-153. We did not observe a statistically significant association between maternal serum CB-153 concentration and infant birth weight, gestational age or risk of prematurity in Kharkiv and Warsaw. It may be stressed, however, that in all cohorts the association was inverse and the adjusted odds ratios of prematurity associated with the increase in log CB-153 concentration were greater than 1.

The results of analysis for p,p'-DDE maternal serum concentration and birth weight and gestational age were more conspicuous than those for CB-153 and we observed statistically significant associations in Inuit and in the Warsaw cohorts. In an unadjusted model increase in log p,p'-DDE concentration was associated with a statistically significant decrease in infant birth weight (Table 4). Control for confounders reduced the effect, but it retained its significance. Adding gestational age reduced the effect by one fourth, nevertheless the effect in Warsaw was significant, and in Greenland was of borderline significance.

**Table 4:** Regression coefficients^1 ^and odds ratios^2 ^(OR) from unadjusted and multivariate linear and logistic regression models of maternal serum log p,p'-DDE and birth weight, gestational age and premature birth

	*Birth weight (grams)*			*Gestational age (weeks)*			*Prematurity*		
	
	Regression	**95% C.I**.	p	Regression	**95% C.I**.	p	OR	**95% C.I**.	p
	coefficient			coefficient					
*Unadjusted^3^model*									
Greenland	-71.4	(-114.7; -28.1)	< 0.01	-0.2	(-0.4; 0.0)	0.02	1.13	(0.87; 1.47)	0.37
Kharkiv	-32.2	(-90.8; 26.4)	0.28	-0.2	(-0.4; 0.0)	0.09	1.43	(0.72; 2.82)	0.31
Warsaw	-131.3	(-231.5; -31.1)	0.01	-0.5	(-0.9; -0.1)	0.02	1.45	(0.73; 2.88)	0.30

*Multivariate model^3,4^*									
Greenland	-56	(-99.5; -12.5)	0.01	-0.2	(-0.4; 0.0)	0.04	1.07	(0.81; 1.41)	0.64
Kharkiv	-48.9	(-109.7; 11.9)	0.12	-0.2	(-0.4; 0.0)	0.06	1.60	(0.75; 3.44)	0.23
Warsaw	-146.9	(-248.8; -45.0)	0.01	-0.6	(-1; -0.2)	< 0.01	2.44	(0.99; 6.06)	0.05

*Multivariate model, with gestational age*									
Greenland	-39.4	(-79.0; 0.2)	0.05						
Kharkiv	-30.5	(-88.7; 27.7)	0.3						
Warsaw	-104.3	(-202.5; -6.1)	0.04						

^1^Per one unit increase of natural log p,p'-DDE

^2^Per twofold increase of untransformed p,p'-DDE

^3^Number of subjects in unadjusted/multivariate models: Greenland birth weight 519/519, gestational age 547/547, prematurity 547/547; Kharkiv birth weight 565/565, gestational age 577/577, prematurity 577/577; Warsaw birth weight 186/186, gestational age 198/198, prematurity 198/198

^4^Models for birth weight controlled for: smoking status (Greenland); age of mother (Kharkiv); age of mother, mother's BMI (Warsaw);

Models for gestational age controlled for: passive smoking (Greenland); mother's age (Kharkiv); mother's education (Warsaw);

Models for prematurity controlled for: mother's education level, mother's age, mother's BMI, marital status, passive smoking (Greenland); mother's age (Kharkiv); education level, smoking status, marital status, mother's age (Warsaw);

The gestational age was significantly reduced when maternal serum p,p'-DDE concentration was increasing in unadjusted and multivariate models in Greenland and Warsaw. In the Kharkiv cohort the gestational age reduction associated with an increase in log p,p'-DDE exposure was of similar magnitude as in the Greenland cohort, however at the borderline of significance.

We observed no significant association between maternal serum concentrations of p,p'-DDE and the risk of premature birth in any study cohort however, all the odds ratios were above 1 and in Warsaw at the borderline of significance.

A very high correlation between the two POP biomarkers in the Inuit study group (Spearman's correlation coefficient r_S _= 0.92) made impossible to disentangle the independent effect of each of them. In Warsaw and Kharkiv the weaker correlation (r_S _= 0.55 and 0.49 respectively) allowed us to run models for birth weight and gestational age with both compounds. Only the association between p,p'-DDE and gestational age in the Warsaw cohort remained statistically significant (-0.7 week decrease, 95% C.I. -1.2 - -0.2, p < 0.01).

## Discussion

Epidemiological evidence regarding adverse effects of POPs on human pregnancy outcome is still limited and results supporting a detrimental association go together with observations that fail to corroborate these results. Our study found a significant reduction in birth weight and length of gestation associated with maternal CB-153 exposure among Inuits but not in the Kharkiv or Warsaw cohorts. However, maternal p,p'-DDE exposure was significantly associated with a reduction in birth weight in the Inuit as well as the Warsaw cohorts, and in the case of gestational age we found the association in all three cohorts (in Kharkiv at the borderline of significance).

Our study found no significant effect of CB-153 and p,p'-DDE on the risk of prematurity however, in all cohorts the OR was above one (in Poland at the borderline of significance). It should be stressed, that small numbers of preterm births, 12 in each of the Warsaw and Kharkiv cohorts and 28 in the Inuit cohort, definitely limited statistical power of our analysis.

As could be expected serum concentrations of CB-153 were much higher in the Inuit mothers as compared with Warsaw and Kharkiv mothers, whereas p,p'-DDE concentrations were highest in the Kharkiv mothers and were rather similar in the Inuit and Warsaw mothers. The exposure pattern in the Kharkiv mothers is in accordance with a previous analysis of maternal milk from Ukraine in which the median p,p'-DDE concentration (2457 ng/g lipid) was one order of magnitude higher than the median CB-153 concentration (149 ng/g lipid) [29]. Concentration levels of CB-153 in the Kharkiv and Warsaw cohorts and of p,p'-DDE in the Inuit and Warsaw cohorts were similar to the concentrations observed in US female population from the National Health and Nutrition Examination Survey in 2003-2004 (median 21.9 ng/g lipid and 207 ng/g lipid for CB-153 and p,p'-DDE, respectively) [30]. It may be of interest to mention that the level of CB-153 concentration in Warsaw and Kharkiv mothers was much lower than observed in the mothers of two districts in neighboring Slovakia (median 140 ng/g lipid) [31].

The possible explanation why the association of CB-153 with birth weight and gestational age was found only among the Inuits may be a much higher exposure level in the Inuit cohort than in the other two. In previous studies, where significant association was observed, the exposure levels due to dietary intakes had been similar or higher than in our Inuit cohort [5-8,13] or were high due to occupational exposure [32], or accidental poisoning [33,34]. On the other hand, however, in some populations, where the exposure levels were similar or higher than among the Inuits in the present study, no significant associations were found [3,9,11,31,35,36]. In a rural Spanish population [10] where the PCB exposure level was similar to that observed in Warsaw and in the population of two Ukrainian cities other than Kharkiv [29] maternal PCB was also not associated with birth weight. Thus, it seems reasonable to conclude that maternal exposure to CB-153 at the levels observed in Warsaw and Kharkiv is not associated with detrimental effects on newborns birth weight, gestational age or risk of prematurity.

Association of maternal DDE exposure with the decrease of birth weight that we observed in the Inuit and Warsaw cohorts corroborates earlier findings in a Great Lakes population study [11], in the Collaborative Perinatal Project (CPP) [15] and in a study in India [37]. On the other hand the lack of association observed in the Kharkiv cohort is in agreement with the results of several other studies that found no association of DDE with birth weight [8-10,16,35,38]. The consistent reverse association of the maternal DDE exposure and the length of gestation that we observed in all three study cohorts (in Kharkiv at the borderline of significance) substantiates the earlier results of the CPP [15].

To take into account the differences in the level of CB-153 as well as p,p'-DDE in the study cohorts we calculated changes in the outcome variables associated with the interquartile change of both POP biomarkers in each study cohort. Such a change is a good measure of the public health importance of the variation in population exposure. An interquartile increase in concentration of CB-153 was associated with the reduction of birth weight by 74.5 g, 8.1 g and 49.4 g, the reduction of gestational age by 0.3, 0.1 and 0.2 weeks and the increase of odds of prematurity by 2.01, 2.26 and 2.85 times in the Inuit, Kharkiv and Warsaw cohorts, respectively. An interquartile increase in concentration of p,p'-DDE was associated with the reduction of birth weight by 47.6 g, 22.9 g and 80.0 g, the reduction of gestational age by 0.3, 0.2 and 0.5 weeks and the increase of odds of prematurity by 1.39, 4.35 and 17.39 times in the Inuit, Kharkiv and Warsaw cohorts, respectively.

The results from the Inuit cohort in Greenland speak in favor of an inverse association between POPs exposure and birth weight and gestational age. However, the high correlation between CB-153 and p,p'-DDE concentrations in serum made it impossible to disentangle independent effects from these compounds. It should also be noted that both CB-153 and p,p'-DDE act as index biomarkers, and other POP compounds could be the ones which affect fetal growth. However, it is of interest to note, that a significant association between p,p'-DDE and a reduction in birth weight and gestational age, was found also in the Warsaw cohort, in which the correlation between CB-153 and p,p'-DDE was only moderate. Moreover, the association in Warsaw was stronger than in Greenland. In the case of gestational age this association retained its significance when we also controlled for CB-153. In Kharkiv both CB-153 and p,p'-DDE were non-significant when simultaneously included in the models. We have not identified a study that would demonstrate an inverse independent association of PCBs and DDE with birth weight or gestational age.

Our results provide only limited evidence that increased CB-153 or p,p'-DDE levels are associated with an increased risk of preterm birth. In all three cohorts and for both chemicals the risks of prematurity were elevated but only for CB-153 concentration in the Warsaw cohort the association reached statistical significance at borderline level. As mentioned above small numbers of preterm births limited statistical power of our analysis. Results of other studies are inconsistent demonstrating presence of significant association [10,15,39] as well as the lack of it [3,16,40,41].

Our study has several strengths. In all participating countries the study was set-up and carried out according to an agreed uniform research protocol regarding measurement of exposure and pregnancy outcome. We used maternal serum CB-153 and p,p'-DDE concentrations as index biomarkers representing direct measurement of POPs as the indicator of intrauterine exposure. Determination of p,p'-DDE and CB-153 in maternal serum was performed for all samples in one specialized center.

On the other hand our study has some limitations as well. The participation rate in the study varied considerably between the populations and while in Greenland we obtained full information from more than 80% of those invited, in Kharkiv the participation rate was only 25%. We have no reason to suspect that the mothers' decision to participate was associated with knowledge of POPs exposure or expected pregnancy outcome in Greenland or in Warsaw. In Kharkiv however, low risk of premature birth (2.1%) could be a result of collecting part of the data (about 12%) at maternity hospitals which were not taking care of mothers with previous preterm birth since such mothers received care at a specialized neonatal hospital. In a study of pregnancy outcome in two urban areas of Ukraine the rate of preterm delivery was 6.6% of singleton live births [42]. This selection bias may be responsible for differing results in the Kharkiv cohort. Also the Warsaw cohort was selected to some extent because it mostly comprised mothers who attended maternity training organized by the hospital. We compared some key variables (birth weight, mothers education, length of gestation, prematurity) in the study cohort and in all births in Warsaw in 2003 that we obtained from the city register. This comparison shows that although our study mothers had on average better education the other variables were not different. We were not able to obtain such data for Kharkiv or Greenland.

## Conclusions

Our results provide some epidemiologic support for an association between *in utero *exposure to the POP biomarkers CB-153 and p,p'-DDE and reduction in birth weight and gestational age however, new insights as to why results vary across studies are not apparent. Because observed birth weight reduction was independent of gestational age our findings suggest detrimental effect of the exposure on intrauterine growth as well as on length of gestation.

## Abbreviations

BMI: body mass index; LOG: natural logarithm; PCB: polychlorinated biphenyls; CB-153: 2,2', 4,4'5,5'-hexachlorobiphenyl; p,p'-DDE: 1,1-dichloro-2,2-bis (*p-*chlorophenyl)-ethylene; DDT: dichlorodiphenyl trichloroetane; POP: persistent organic pollutant; EDF: effective degrees of freedom.

## Competing interests

The authors declare that they have no competing interests.

## Authors' contributions

JPB, LH designed and initiated Inuendo project. GT, HSP, JKL, KG, VZ and AL were responsible for collecting the blood samples and the interview data. BAGJ was responsible for the chemical analysis of the POP biomarkers. JPB and GT coordinated the execution of the Inuendo project. GT has main responsibility for creating Inuendo database. BJW initiated the newborn study. DR and BJW had main responsibility for creating newborn database. BJW and DR were responsible for statistical analysis and writing the draft version of manuscript. All authors commented on and approved the final manuscript.
